# Systematic Comparative Evaluation of Methods for Investigating the TCRβ Repertoire

**DOI:** 10.1371/journal.pone.0152464

**Published:** 2016-03-28

**Authors:** Xiao Liu, Wei Zhang, Xiaojing Zeng, Ruifang Zhang, Yuanping Du, Xueyu Hong, Hongzhi Cao, Zheng Su, Changxi Wang, Jinghua Wu, Chao Nie, Xun Xu, Karsten Kristiansen

**Affiliations:** 1 BGI-Shenzhen, Shenzhen, 518083, China; 2 Shenzhen Key Laboratory of Transomics Biotechnologies, BGI-Shenzhen, Shenzhen, 518083, China; 3 Department of Biology, University of Copenhagen, Copenhagen, Denmark; Cornell University, UNITED STATES

## Abstract

High-throughput sequencing has recently been applied to profile the high diversity of antibodyome/B cell receptors (BCRs) and T cell receptors (TCRs) among immune cells. To date, Multiplex PCR (MPCR) and 5’RACE are predominately used to enrich rearranged BCRs and TCRs. Both approaches have advantages and disadvantages; however, a systematic evaluation and direct comparison of them would benefit researchers in the selection of the most suitable method. In this study, we used both pooled control plasmids and spiked-in cells to benchmark the MPCR bias. RNA from three healthy donors was subsequently processed with the two methods to perform a comparative evaluation of the TCR β chain sequences. Both approaches demonstrated high reproducibility (R^2^ = 0.9958 and 0.9878, respectively). No differences in gene usage were identified for most V/J genes (>60%), and an average of 52.03% of the CDR3 amino acid sequences overlapped. MPCR exhibited a certain degree of bias, in which the usage of several genes deviated from 5’RACE, and some V-J pairings were lost. In contrast, there was a smaller rate of effective data from 5’RACE (11.25% less compared with MPCR). Nevertheless, the methodological variability was smaller compared with the biological variability. Through direct comparison, these findings provide novel insights into the two experimental methods, which will prove to be valuable in immune repertoire research and its interpretation.

## Introduction

T cells and B cells are the most important components of the adaptive immune system. These cells enable the host to resist a vast array of potential pathogens via the production of diverse T cell receptor (TCR) or B cell receptor (BCR)/immunoglobulin (Ig) repertoire. The nucleotide sequences that encode the TCR or BCR are produced via somatic rearrangement of Variable, Diverse (D) and Joining (J) segments, as well as a set of non-template nucleotide insertions and deletions at the V-(D)-J junction region. Together, these processes contribute to the generation of diversity in TCRs or BCRs. Most of the variation in each chain is located in complementary-determining region 3 (CDR3), which is encoded by the V(D)J junction. The CDR3 of the TCR β-chain (TRB) region, defined by the IMGT collaboration [1], begins with the second conserved cysteine encoded by the 3’ portion of the V gene segment and ends with the conserved phenylalanine encoded by the 5’ portion of the Jβ gene segment. Besides, Ig introduces point mutations during the process of affinity maturation of B-cells in the germinal center, which further increases the diversity of the Ig repertoire. Theoretically, the potential diversity of TCRs-αβ (TCR α chain and β chain) may attain 10^18^ for humans according to the combinatorial mechanism, whereas the potential diversity of the B-cell repertoire is even higher considering the somatic hyper-mutation (SHM)[2, 3]. Wang *et al*. estimated a sample with 0.47×10^6^ TCR-α (TRA) unique nucleotide sequences and 0.35×10^6^ TCR-β unique CDR3 nucleotide sequences [4]. Jacob Glanville *et al*. estimated that a human donor has at least 3.5×10^10^ unique IgM sequences using a capture-recapture method [5].

As a result of the high diversity of the immune repertoire, the limited output of Sanger sequencing and the low resolution of GeneScan provide only limited visualization of this process. The next generation sequencing (NGS) platforms, such as Roche 454 and Illumina Hiseq, are ideally suited to derive a holistic snapshot of the process and extensively characterize and visualize the complexity and plasticity of the TCR and BCR repertoires [4, 6–9].

Irrespective of the sequencing platform, it is imperative to enrich the V(D)J rearranged genomic or transcript sequences prior to sequencing, using methods such as multiplex PCR (MPCR), Rapid Amplification of cDNA Ends (5’RACE), linear PCR or sequence capture. He *et al*. have used a sequence capture method denoted IgCap to enrich the somatically rearranged DNA sequences from lymphomas [10]. However, IgCap captures all fragments that contain relevant Ig heavy chain (IgH) gene sequences, whereas it does not simply capture the rearranged fragments; thus, it provides limited sensitivity and resolution. The linear amplification method begins with the V gene primer set, followed by the C (constant)-gene primers in the second direction extension [11]. It requires more starting material and has less amplification bias, in theory. However, the strong bias of linear amplification was also observed in our test (data not shown).

Of all of these methods, MPCR and 5’RACE are the two most commonly used approaches because of their obvious advantages compared with the other approaches. MPCR utilizes a pool of primers that targets all V and J germline genes (or C genes) to amplify the whole V(D)J rearrangements or specifically the CDR3 regions [8, 12–14]. Although MPCR may cause PCR bias because of the inconsistent reaction efficiencies of multiple primers [2], it requires only one PCR step prior to library construction and is suitable for both genomic DNA and RNA materials, which makes it a convenient and cost effective approach.

The 5’RACE is another commonly used method [7, 15, 16] for investigating the immune repertoire. It only utilizes one primer set that targets the known C-gene region of the mRNA transcripts. The second general primer is synthetically added to the 3' end of the cDNA using terminal deoxynucleotidyl transferase (TdT) and cCTP. This method introduces negligible PCR bias compared with other methods [2] although it may only be used for RNA samples.

**Fig 1:**
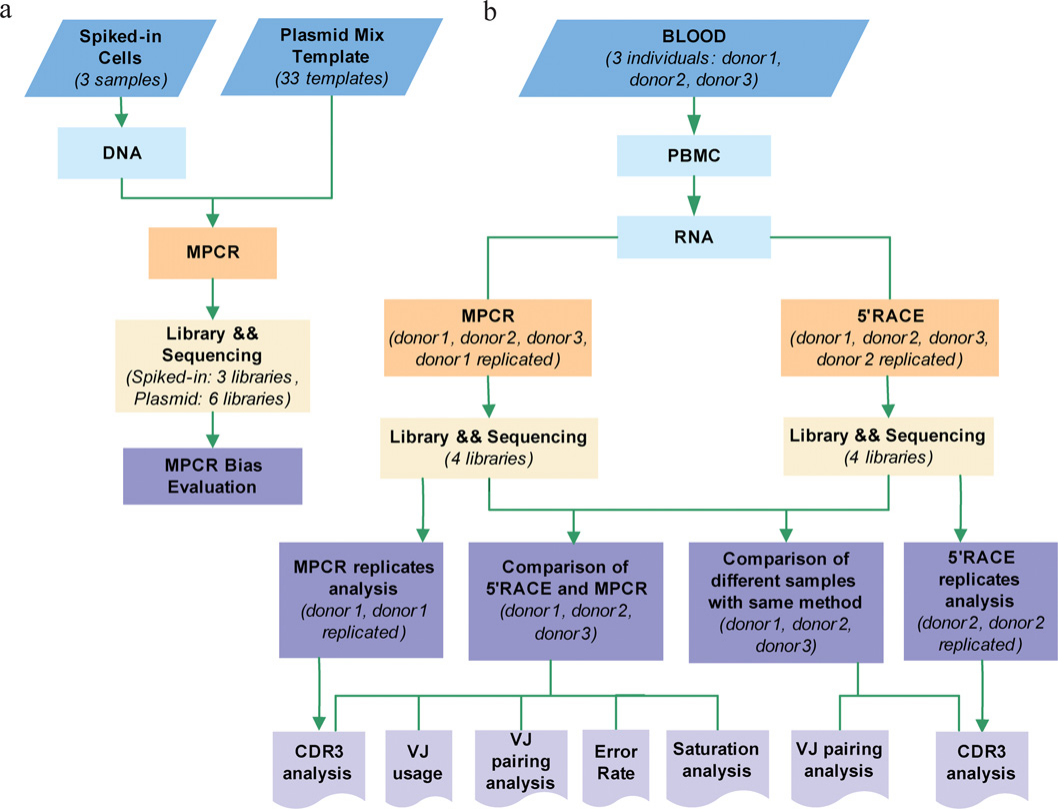
Overview of project design. (**a**), MPCR bias was evaluated using three T cell populations spiked with cells of known TCRs and three controls (with replication) pooled with 33 plasmids inserting known TCR sequences. (**b**), Comparison of MPCR and 5’RACE. RNA was derived from the PBMC of three healthy donors. It was used to profile the TRB repertoire via both MPCR and 5’RACE. The analyses included a calculation of MPCR/5’RACE reproducibility and comparison of the repertoire characteristics between 5’RACE and MPCR.

Despite their wide-ranging applications demonstrated by various studies, a direct and systematic comparative evaluation between these two methods is lacking. Benichou *et al*. have provided comments regarding published papers [2]; however, limited head to head comparisons of the two methods have been published. In this study, we designed experiments to comparatively evaluate these two methods. First, pooled-plasmid controls and cell populations spiked with known TCRβ rearrangements were designed to benchmark the MPCR bias (Fig 1A); limited bias was identified. Second, we simultaneously performed MPCR and 5'RACE using identical RNA samples extracted from the peripheral blood of 3 individuals to profile the TCRβ repertoire. We also prepared a replicate for MPCR and 5’RACE (Fig 1B). We compared the effective data, V and J gene usage, sequence diversity, and V-J pairing, in addition to the level of clonal sequences. Finally, saturation analyses were conducted for different samples (Fig 1B). Overall, the respective features of these two methods provide deeper insights in the investigation of the immune repertoire.

## Materials and Methods

### Blood Samples

Samples of peripheral blood mononuclear cells (PBMC) from 2 healthy men and 1 healthy woman (S01, S02, and S03) aged 22 to 34 years were obtained with written informed consents. PBMCs were immediately isolated from each sample using Ficoll-Paque (GE Healthcare) gradient centrifugation. RNA was isolated using Trizol (Invitrogen) according to the manufacturer’s specifications. The RNA concentration and sample integrity were determined on an Agilent Bioanalyzer (Agilent).

### Plasmid Pools

Thirty-three plasmid clones were selected from the TA-cloning library of one TCR β-chain repertoire. Each plasmid was cloned with a specific TCRβ CDR3 region sequence. All TCRβ V and J subfamilies were included in the plasmid pools. One plasmid pool was prepared with equal moles of each plasmid, and the other two plasmid pools were prepared with different pooling ratios (ratios of 1:10, 1:100, 1:1000, and 1:10000). All three pools were replicated. The details regarding the different pooling ratios are shown in S3 Table.

### Spiked-In Samples

Spiked-in samples were generously donated by professor Karen Cerosaletti [17]. In brief, five CD4+ T cell clones, previously reported as specific for GAD65, which had unique TCRβ CDR3 sequences, were spiked into CD4+CD45RA+ naïve T cells sorted from the fresh PBMC of a control donor. Three CD4+T cell mixes, which contained different amounts of the five CD4+ T cell clones as measured via flow cytometry, were prepared. In mix 1, 10^5^, 10^4^, 1000, 100 and 10 T cells of the five clones were doped in a background of 1 million naïve T cells. In mix 2, the same 1000 cells for each clone were doped, and 10, 100, 1000, 10^4^ and 10^5^ were doped in mix 3. gDNA and total RNA were extracted from the cells using commercially available kits. The details of the five clones are shown in S4 Table.

### MPCR Method

We used MPCR primers from a published paper [18] to amplify the rearranged CDR3 regions of TCRβ, including 30 forward V primers and 13 reverse J primers. One to 3 μg of total PBMC RNA was subjected to DNase I digestion (NEB) for 10 min at 37°C to remove genomic DNA. The digestion reaction was terminated via the addition of 2 μL 1 mM EDTA for 10 min at 75°C. The product was subsequently purified via ethanol precipitation. First strand cDNA was synthesized using SuperScript Ⅱ reverse transcriptase (Invitrogen) with a primer specific to the TCRβ constant region (5’>CACGTGGTCGGGGWAGAAGC<3’). First-strand cDNA was used as a template for multiplex PCR with an equimolar pool of the 30 TCR Vβ forward primers (the VβF pool) and an equimolar pool of the 13 TCR Jβ reverse primers (the JβR pool). The primer set is available upon request. The reaction conditions were as follows: 1×QIAGEN Multiplex PCR Master Mix, 0.5×Q solution, 0.2 μM VβF pool and JβR pool in a 50-μL volume. The cycling conditions were as follows: 95°C for 15 min, 25–30 cycles at 94°C for 30 sec, 60°C for 90 sec, and 72°C for 30 sec, plus a final extension at 72°C for 5 min. The PCR product was run on a 2% agarose gel; the band size between 110–180 bp was excised from the gel and purified.

### 5’-RACE Method

5’-RACE was performed using the 5’ RACE System for Rapid Amplification of cDNA Ends, Version 2.0 (Invitrogen). First-strand cDNA was synthesized using a published TRBC (TRB C region) primer (5’>CACGTGGTCGGGGWAGAAGC<3’). The reaction conditions were as follows: 3–5 μg RNA, TRBC primer 2.5 pmoles, 1×PCR buffer, 2.5 mM MgCl2, 0.4 mM each dNTP, 10 mM DTT, and 200 units of Superscript II in a 25 μL volume. Extension occurred at 42°C for 50 min, followed by inactivation at 70°C for 15 min; 1 μl of RNase mix was subsequently added, followed by incubation at 37°C for 30 min to remove RNA. The cDNA was then purified with a S.N.A.P. Column. The purified cDNA was subsequently used in the TdT-tailing reaction to add an oligo-dC tail in the 3’ end of cDNA using TdT and dCTP. The dC-tailed cDNA was directly amplified via PCR. PCR was performed using Taq DNA polymerase (TAKARA) in a 50 μL volume with a deoxyinosine-containing abridged anchor primer (AAP) provided with the 5’ RACE system (5’>GGCCACGCGTCGACTAGTACGGGIIGGGIIGGGIIG <3’) and an equimolar combination of two biotinylated GSPs, (5’> ACACTTAATTAACGGGTGGGAACACCTTGTTCAGGT<3’) and (5’>ACACTTAATTAACGGGTGGGAACACGTTTTTCAGGT<3’), which contain 5’ PacI sites and were specific for TRBC1 and TRBC2, respectively[15]. The reaction conditions were as follows: 1×PCR buffer, 1.5 mM MgCl2, 0.2 mM each dNTP, 0.4 μM AAP, 0.4 μM biotinylated GSP, 5 μL dC-tailed cDNA, and 2.5 units Taq DNA polymerase. A 2 min denaturation at 94°C was followed by 30 cycles of 30 sec at 94°C, 30 sec at 55°C and 45 sec at 72°C, as well as a final extension at 72°C for 5 min. The PCR product was run on a 2% agarose gel; the band centered at 520 bp was excised and purified. Approximately 3 micrograms of purified PCR product were subsequently sheared using a Covaris S2 (Applied Biosystems). The parameters of the reaction conditions were as follows: 100 μL reaction volume, 12 cycles with a duty cycle of 10%, intensity of 5, and cycles per burst at 200 for 30 sec. Biotinylated fragments were then purified using 50 μL of Dynabeads M-270 Streptavidin (Invitrogen) prepared according to the manufacturer’s specifications. Washed and bound biotinylated fragments were subsequently cleaved with PacI [reaction conditions; 1× NEB buffer 1, 1× BSA, and 10 U PacI (NEB) in a 50 μL volume for 1 h at 37°C, followed by 20 min at 65°C]. The sample was run on a 2% agarose gel; the fraction from 150–190 bp was excised and purified.

### Library Preparation and High Throughput Sequencing

The MPCR and 5’-RACE products were prepared for Hiseq 2000 sequencing according to the manufacturer’s protocol with modifications. The end repair was performed using T4 DNA polymerase, Klenow fragment of *E*.*coli* DNA polymerase I and T4 PNK in T4 PNK buffer (all Enzymatics) at 20°C for 30 min. Following a Qiaquick PCR purification (Qiagen), a deoxynucleotides were added to polished double strands using the Klenow fragment (3’-5’-exo-) in the presence of 200 μM dATP in 1×blue buffer (all Enzymatics) at 37°C for 30 min. Following MinElute PCR purification (Qiagen), Illumina PE adaptors were ligated. The reaction conditions were as follows: 1×Rapid ligation buffer, 3000 U T4 DNA ligase (Rapid) (all Enzymatics), and 0.8 μM PE adapters in a 50 μL volume at 20°C for 15 min. The product was purified using 1.2 times the volume of Ampure XP (AGENCOURT) and eluted in a volume of 23 μL. The ligation product was subsequently amplified via PCR using Phusion HF Master mix (Finnzymes) with 0.2 μM of Illumina primers 1.0 and 2.0 in a 50 μL volume. The cycling conditions were as follows: a 2-min denaturation at 98°C was followed by 12 cycles of 20 sec at 98°C, 30 sec at 65°C, and 30 sec at 72°C, as well as a final extension for 5 min at 72°C. The PCR product was purified using 1.2 times the volume of Ampure XP and eluted in a volume of 15 μL. Libraries with insert sizes of 100–200 bp were analyzed via Bioanalyzer analysis and QPCR and were stored at -20°C until use. Paired-end 100 bp sequencing was performed using an Illumina Hiseq 2000 system following the manufacturer’s instructions. Fluorescent images were processed into sequences using the Illumina data processing pipeline.

### Data Analysis

Sequencing data were analyzed using IMonitor [18], which is briefly introduced as follows. First, it performs basic QC and filters the low quality reads of NGS data; it subsequently merges the cleaned pair end reads to be connected. Second, the merged data are used to perform a BLAST [19–21] alignment to the V, D, J germline genes and alleles (IMGT, http://www.imgt.org/), respectively. After acquiring the BLAST alignment results, it performs a re-alignment for each result and selects the best V/D/J alignment for each merged read. Third, it filters the sequences in which the abundance (support reads) of the corresponding CDR3 nucleotide sequence is less than 5 and translates these sequences to protein sequences. Finally, it performs multiple statistics of the TCR data, such as V-J pairing, V/J usage, CDR3 sequence frequency statistics, and CDR3 length distribution.

### Statistical Analysis

We used Pearson correlation coefficients[22] to measure the linear correlation between two variables. The analysis included MPCR and 5’RACE reproducibility evaluation (observed frequencies and expected frequencies), as well as the evaluation of the similarity of two samples using CDR3 frequencies. The statistical significance of the difference in the V/J frequency between the MPCR and 5’RACE samples was determined using paired t tests. The Chao1 has been used to estimate the target richness for individual-based data for ecology. Here, we used this method to estimate the maximum number of unique CDR3 amino acid sequences of the samples. Estimated values using the Chao1 algorithm and observed values were used to create the saturation curves [23, 24]. The Chao1 bias corrected method was quoted, and the formula was as follows:

$${\hat{S}}_{chao1}=S_{obs}+\frac{F_{1}{\left(F_{1}-1\right)}^{}}{2(F_{2}+1)}$$ (1)

S_obs_ represents the total number of observed clono-types in a sample; *F* denotes the number of clono-types (*F*_1_, the number of clono-types detected one time. *F*_2_, the number of clono-types detected two times).

This research was reviewed and approved by a duly constituted ethics committee (the Institutional Review Board on Bioethics and Biosafety of BGI).

## Results

### MPCR Bias Evaluation

#### Plasmid pools

**Fig 2:**
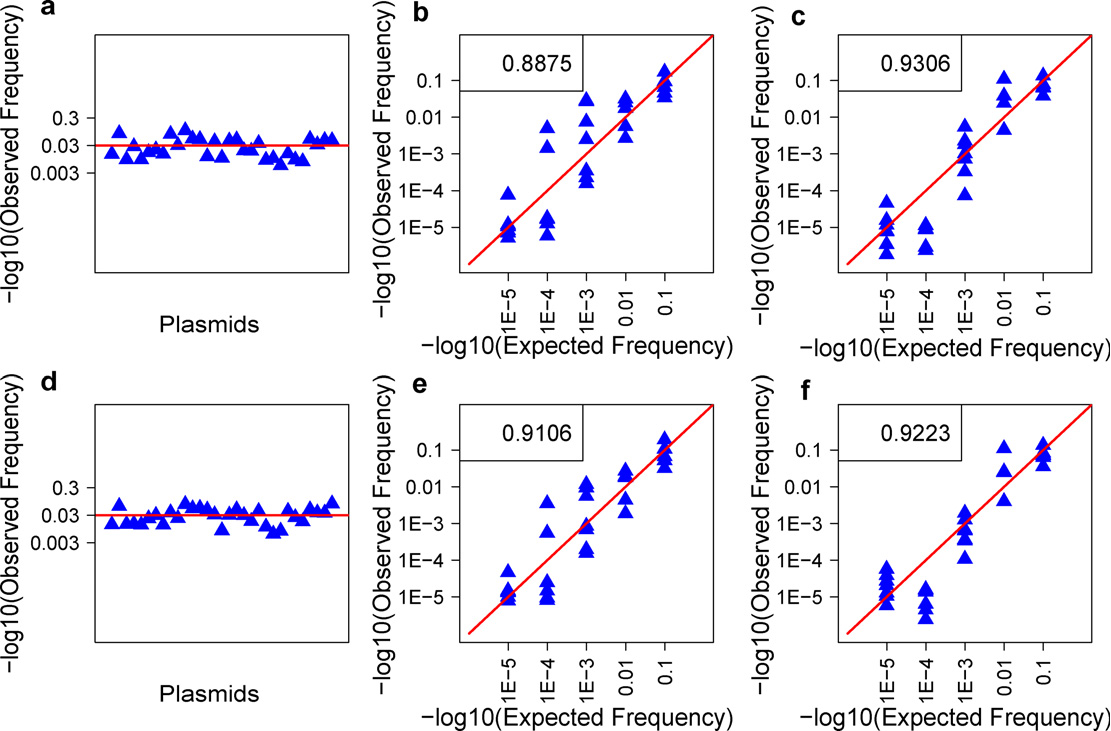
MPCR bias evaluated by the mixed plasmid samples. Thirty-three plasmids were mixed in the sample with three different pooling gradients (a, b, and c represent plasmid mixes 1, 2, and 3, respectively, details in S3 Table). Their replications are showed in d, e, and f. The observed frequencies of the thirty-three templates were calculated and compared with the expected pooling frequencies. The numbers in the top left corner (**b, c, e,** and **f**) represent the Pearson correlation coefficients between the observed and expected frequencies.

One major concern for MPCR is its potential bias towards specific clones because of the inconsistent amplification efficiencies among the multiplex primers. To evaluate this issue, three-plasmid pools, one pool with an equal mole of each plasmid and the other two pools with different pooling ratios (S3 Table), were used to compare the expected and observed frequencies of the inserted TCRβ CDR3 sequences. Each plasmid pooling experiment was replicated (Fig 2). The plasmid clones C-16 and C-17 were removed in the subsequent analysis because nearly none of corresponding reads were identified in all six pools. An average Pearson R^2^ (correlation coefficient) of 0.9247 was identified between the replications, which demonstrated an excellent stability for MPCR. Although some observed frequencies did not exactly match the expected pattern, the differences were not significant in all six pools. The average high correlation coefficient (R^2^ (average) = 0.9130) between the observed and expected frequencies demonstrated an overall low bias for MPCR in these plasmid-mix samples.

#### Spiked-in samples

**Fig 3:**
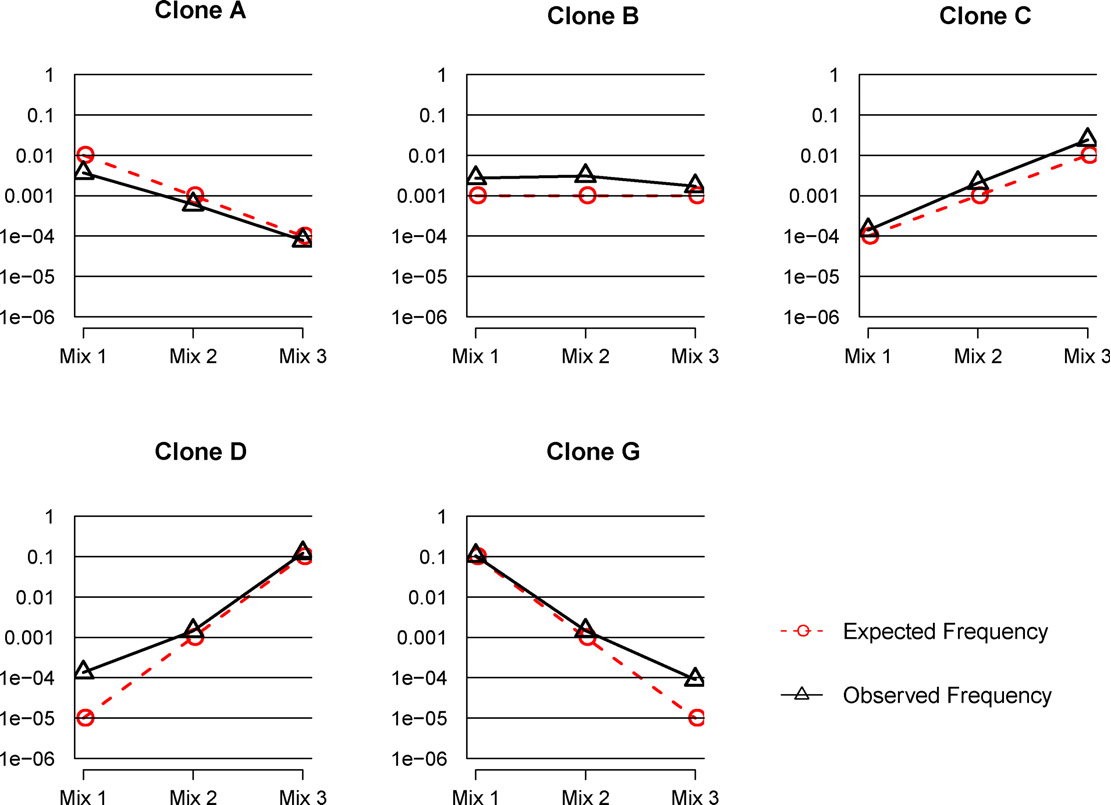
Expected versus observed frequencies for 5 spiked-in clones in three mixtures. **F**ive CD4+ T cell clones (A, B, C, D, and G) were spiked into sorted CD4+ T cell populations at concentrations that spanned five orders of magnitude. TRB CDR3 was amplified via MPCR, and the observed clone frequency was calculated. The results demonstrated a high concordance between the expected and observed clone frequencies in three samples (Mix1, Mix2, and Mix3).

Three spiked-in T cell populations with defined ratios of T cells of known TCR sequences were also adopted to evaluate MPCR. gDNA was extracted, amplified with MPCR, and sequenced for an average of 10 million TCR β sequences (S4 Table). Overall, the five spiked-in clones were identified in each of the three samples at levels comparable to the expected frequencies (Fig 3). Specifically, clones A and C exhibited high concordance between the expected and observed frequencies in all three mixtures; clone B was slightly over amplified; clones D and G were nearly identical with the expected frequencies of 1:10 and 1:10^3^ ratios; however, they were over-represented in the mixture of the 1:10^5^ ratio. Furthermore, in the spiked-in experiment, 10 clone cells of one million were identified, which illustrated the high sensitivity of the MPCR method to track low frequency or rare clones.

### Effective Data

RNA was extracted from the PBMC of three healthy donors and was subsequently divided into two parts to amplify the TCR β region. Together, 4 MPCR libraries and 4 5’RACE libraries, including the replicates, were sequenced with paired-end 100 bp using a Hiseq2000 (Illumina Inc.) The data statistics are shown in Table 1. Each sample was sequenced for 8–46 M paired-end reads. The insert sizes of some 5’RACE fragments were either too short (less than 100bp) or too long (more than 200bp) because of random shearing. The fragments less than 100bp were easily polluted by sequencing adapter, so the filtered data of 5’RACE samples (22.05% on average) were more than that of MPCR samples (2.69% on average). The fragments longer than 200bp could not be merged, so less sequences were merged for 5’RACE samples. Furthermore, the VJ alignment rate of each MPCR sample was greater than 98%, whereas it was less than 79% for each of the 5’RACE samples. It was because the sequences of 5’RACE samples included an approximately 30 bp C region, which left some sequences with very short or even no V gene nucleotides for identification. Most sequences were identified the correct ORF (open reading frame) for all samples. To decrease the sequencing errors, the sequences detected for less than 5 times (5 reads supported) were filtered. Overall, the average effective data rate for MPCR was 80.98%, whereas it was 47.48% for 5’RACE. With the exception of S02-R-2, the number of unique CDR3 nucleotide and amino acid sequences were all greater than 10^5^, and its number of S03-M and S03-R were several times more than the other samples because they were started with more cells and RNA amounts. Simpson’s diversity index [25] of the CDR3 AA (amino acid) sequences was calculated to quantify the distribution of diversity in each sample (Table 1).

**Table 1:** Statistics of the multiplex PCR and 5’RACE libraries.

Sample	S01-M-1	S01-M-2	S02-M-1	S03-M-1	S01-R-1	S02-R-1	S02-R-2	S03-R-1
**Total reads**	8,126,815	14,588,545	12,612,786	39,754,077	24,555,544	24,450,175	24,204,014	46,409,300
**Clean reads**^a^	7,955,514	14,293,710	12,396,695	37,797,350	18,559,742	18,590,382	17,739,268	40,325,274
**PE reads merged**^b^	7,934,499	14,251,076	12,365,441	37,447,896	16,755,290	17,713,913	15,704,110	37,861,325
**VJ alignment**^c^	7,821,564	14,054,378	12,150,255	36,496,389	10,763,131	13,726,534	12,427,498	29,851,422
**Correct ORF reads**^d^	7,439,792	13,448,043	11,122,904	34,838,414	9,585,671	12,638,442	11,460,936	28,265,147
**Effective reads**^e^	6,880,594	12,664,905	10,398,513	27,823,441	9,585,671	12,019,778	11,023,329	26,067,794
**Effective reads Rate**	84.67%	86.81%	82.44%	69.99%	39.04%	49.16%	45.54%	56.17%
**Unique CDR3 nucleotides**^f^	148,262	204,041	170,733	1,061,044	265,458	156,655	81,008	541,446
**Unique CDR3 peptides**^g^	138,831	188,164	158,991	858,389	247,403	147,035	76,636	483,083
**Simpson’s diversity index (CDR3)**	0.9961	0.9949	0.9288	0.9878	0.9881	0.9985	0.9985	0.9828

^a^Clean reads refer to reads after filtering the low-quality sequences

^b^PE merged reads refer to paired-end reads merged filtered by the sequences with a stop codon, incorrect CDR3 length, and mapped to pseudo genes

^c^VJ alignment refers to reads mapped to V and J genes

^d^Correct ORF reads were determined to have the CDR3 region

^e^Effective reads were filtered by the low-frequency sequences

^f^Unique CDR3 nucleotide represents a non-redundant fragment (CDR3) of nucleotide

^g^Unique CDR3 peptide represents a non-redundant fragment (CDR3) of amino acids.

### Reproducibility of MPCR and 5’RACE

To investigate the reproducibility of MPCR and 5’RACE, two separate aliquots (S01-M-1 and S01-M-2) of RNA from individual S01 were used to prepare MPCR sequencing libraries, whereas S02-R-1 and S02-R-2 from S02 were used for 5’RACE. All subsequent analyses were based on the same amount of effective reads for the replicated samples. Specifically, effective data were randomly sub-sampled to 6,880,594 sequences per library for the MPCR method and 11,023,329 sequences per library for the 5’RACE. The basic statistics are shown in S1 Table.

**Fig 4:**
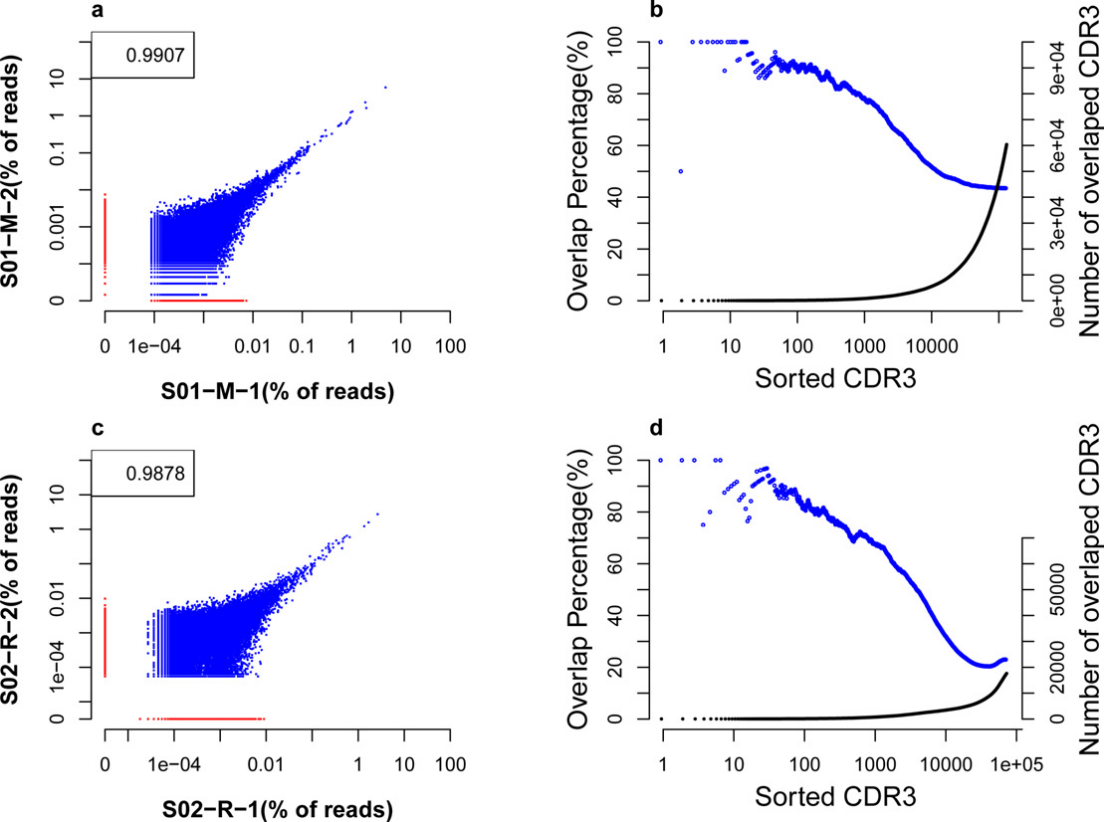
Correlation and consistency in MPCR/5’RACE replicate samples. (**a**) The correlation of the CDR3 AA frequencies in two MPCR replicates. Each dot represents a unique CDR3 AA sequence. Shared CDR3 sequences are indicated in blue, and specific sequences are indicated in red. The number in the top left corner is the Pearson correlation coefficient of the two data sets. (**b**). The consistency of the CDR3 sequences between two MPCR replicates. CDR3 AA sequences are ranked by frequencies in each replicate. We subsequently compared the shared CDR3s between the two replicates beginning with the top ranking clones, e.g., top 10 and top 100. The number (right Y axis) and percentage of shared sequences (left Y axis) are calculated and shown in the solid black dot and blue circle, respectively. (**c**). The correlation of the CDR3 amino acid sequences in two 5’RACE replicates. (**d**). The consistency of the CDR3 sequences between two 5’RACE replicates.

For MPCR, the data analysis indicated that the number of unique CDR3 was similar between the two replicates. In contrast, the two replicates of 5’RACE exhibited a greater difference (S1 Table), which may be attributed to the variable fragmentation in the 5’RACE experiment. The replicated samples for MPCR shared 69,634 unique CDR3 amino acid (AA) sequences, which corresponded to 78.63% and 74.93%, respectively, of their total sequences. For 5’RACE, 25,773 unique CDR3 AA sequences were shared, which represents 50.01% and 59.68%, respectively, of the total reads. Moreover, both MPCR and 5’RACE exhibited high reproducibility for CDR3 AA abundance, with Pearson correlation coefficients of 0.9907 for MPCR and 0.9878 for 5’RACE (Fig 4A and 4C).

To further evaluate the reproducibility, we calculated the consistency between the replicated samples. CDR3 AA sequences were ranked by frequencies in each replicate; we subsequently calculated the shared CDR3 number (accumulative shared number) and percentage (accumulative percentage) between the two replicates beginning with the top ranking clones, e.g., top 10 and top 100. For example, ranked CDR3 1000 (at x axis in Fig 4B) indicates 78.5% CDR3s were shared among the top 1000 CDR3s. The shared percentage of replicates slowly decreased along with the decrease in the CDR3 abundance for both MPCR and 5’RACE. In MPCR, the top 19 CDR3 sequences of the two libraries were identical with the exception that some sequences ranked differently between the two replicates. For the top 1000 sequences, up to 78.5% of the sequences were shared; While overall, approximately 43.48% of the unique sequences were shared (Fig 4B). In 5’RACE, there were 96% identical sequences in the top 30 CDR3 sequences of the two replicates, 68% in the top 1000, and approximately 22.96% overall were shared (Fig 4D). Overall, the reproducibility of MPCR and 5’RACE was very high, especially for MPCR.

### V and J Gene Usage Comparison between MPCR and 5’RACE

**Fig 5:**
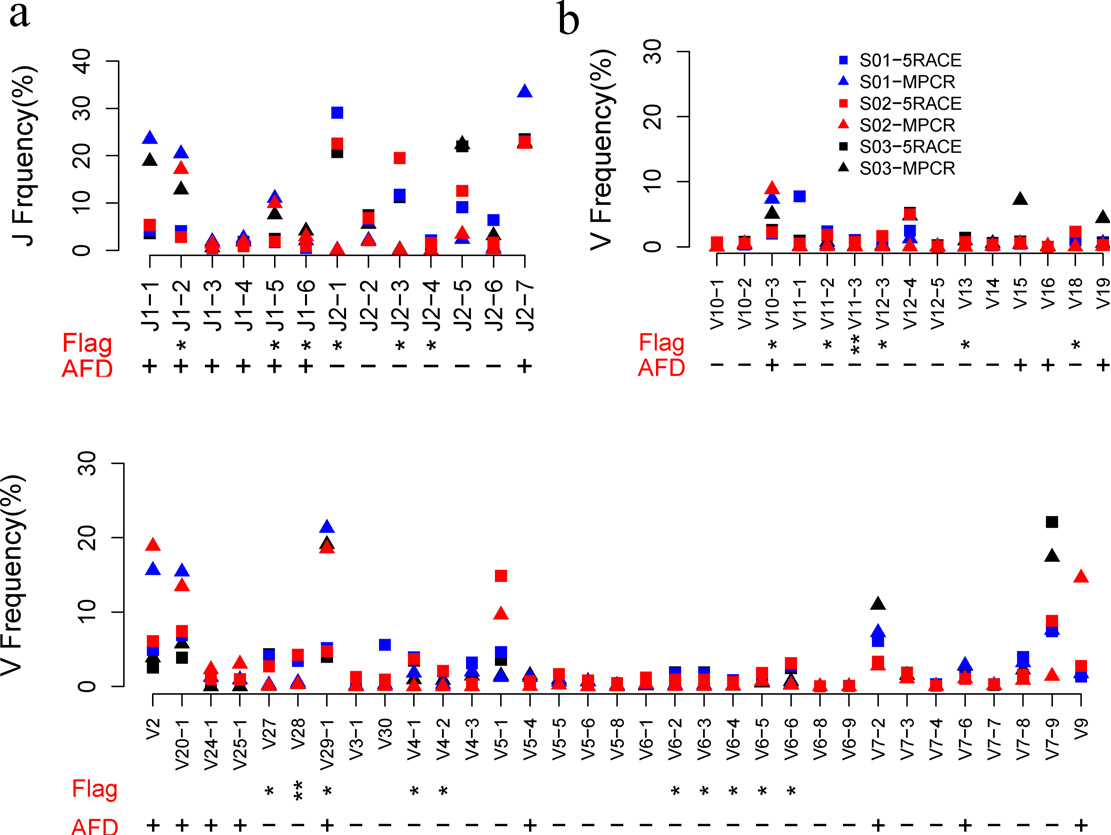
Frequency distribution of TRBV and TRBJ genes for both MPCR and 5’RACE samples. TRB of three samples were amplified by both MPCR (trilateral) and 5’RACE (square). All V and J frequencies were normalized. The V and J frequencies were compared between the MPCR and 5’RACE samples. Flag: *, p-value < 0.05. **, p-value <0.01, paired t test. AFD, average frequency difference between the 5’RACE and MPCR samples (AFD = f(MPCR)-f(5’RACE)). +, AFD>0; -, AFD<0. (**a**) Frequencies of 13 TRBJ genes. (**b**) Frequencies of 48 functional TRBV genes.

There are 48 functional V genes and 13 J genes for the TRB, which contribute to the high diversity in the CDR3 region. In this study, the V and J usage of six samples were analyzed. The results are shown in Fig 5. Furthermore, the average frequency difference (AFD) between the 5’RACE and MPCR samples was calculated. Paired T tests were used to determine the difference between the two methods.

The usage of most V and J gene exhibited similar patterns without significant differences, including 32 V genes (66.67%) and 7 J genes (53.85%), indicating no overall significant bias in the MPCR method. However, several genes, such as TRBV29-1 and TRBJ2-1, exhibited a usage variance between the two methods (p<0.05, paired t test). Among these genes, 3 J had a higher frequency in MPCR compared with 5’RACE(AFD>0), whereas the majority of V genes (14/16 or 87.5%) exhibited a lower frequency in the MPCR method (AFD<0). This finding may be a result of the lower kinetics efficiency during the amplification process for primers that target these genes. Furthermore, we determined that the overall 72.92% (35/48) V gene frequency of MPCR was less than 5’RACE (AFD<0, Fig 5), which was accounted for the bias of several abundant genes, such as TRBV29-1 and TRBV20-1.

### CDR3 Sequence Comparison between MPCR and 5’RACE

**Fig 6:**
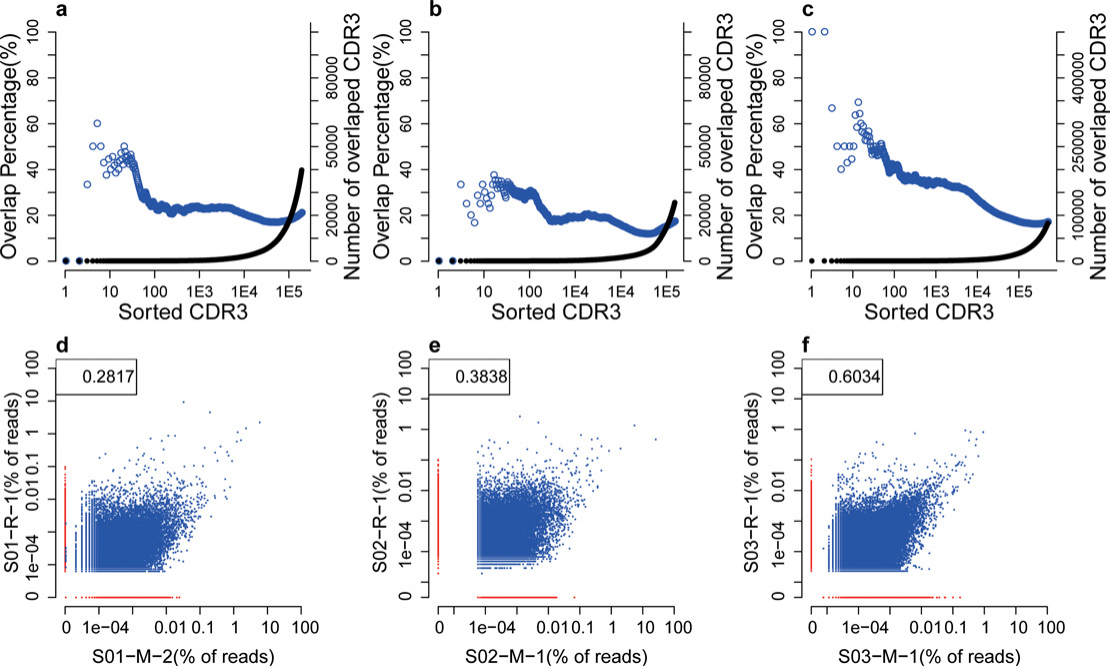
Correlation and consistency between MPCR and 5’RACE samples. (**a, b, c**), the consistency of S01, S02, and S03. CDR3 AA sequences are ranked by frequencies in each replicate. We subsequently compared the shared CDR3s between the two replicates beginning with the top ranking clones, e.g., top 10 and top 100. The number (right Y axis) and percentage of shared sequences (left Y axis) are calculated and shown in the solid black dot and blue circle, respectively. (**d, e, f**), the correlations among S01, S02, and S03. The blue points represent the shared CDR3 in both samples; the red points represent the specific CDR3 in each sample. One extreme point was removed from S02 and S03. The number in the top left corner is the Pearson correlation coefficient of the two data sets.

The variations associated with specific V and J genes were identified in this study; however, it was unclear how this bias may impact CDR3 sequences. To investigate this issue, the CDR3 AA sequences were sorted by frequencies in descending order for the MPCR and 5’RACE data sets (Fig 6A, 6B and 6C). The consistencies of the two sorted data sets produced by the two different experimental methods were calculated. For the 3 individuals, the sequences in the top 100 unique CDR3 amino acid sequences of the two libraries had concordances of 24.0%, 30.0% and 40.0%, respectively. The overlapping rates of the unique CDR3 sequences were 21.07%, 17.35% and 17.07% for individual S01, S02 and S03, respectively, based on the entire dataset. S01 had a lower rate in the top 100; however, its overall rate was 4% higher compared with the other individuals, implying the sharing of identical sequences in lower frequency for the two methods.

The overlap rate in unique CDR3 AA sequences was approximately 20%; however, the overlap rate increased to an average of 55.42% when their abundances were considered (S2 Table). In particular, the overlap rates were 59.40% and 54.10%, respectively, for S01, 66.32% and 43.03%, respectively, for S02, and 55.98% and 53.69%, respectively, for S03. Thus, abundant clonotypes had a higher consistency in frequency between the two methods. The correlation was also calculated from 3 individuals (Fig 6D, 6E and 6F). The coefficient of the correlation in the abundance of CDR3 AA sequences between MPCR and 5’RACE sequencing for each individual ranged from 0.2817 to 0.6034 (0.4230 on average).

### V-J Pairing Comparison between MPCR and 5’RACE

**Fig 7:**
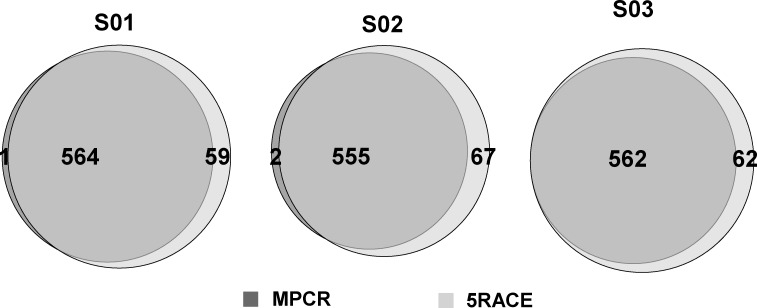
Comparison of V-J pairing coverage between MPCR and 5’RACE. Light gray represents the number of covered V-J pairings for 5’RACE samples; dark gray represents the number for MPCR samples.

When we examined the V-J gene pairing covered by each sample, we determined that the 5’RACE samples contained nearly all 624 potential V-J pairs (48 TRBV * 13 TRBJ), whereas the MPCR samples, on average, lost 63 pairs (Fig 7). On average, the overlapping V-J pairing number for each sample was 560 (89.80%). The more diversified pattern of V-J pairing identified by the 5’RACE method indicated that it introduced less bias compared with MPCR. Overall, the correlation coefficient of V-J pairing abundance between the two methods ranged from 0.2777 to 0.7202.

### Method Variation in Comparison to Biological Variation

The direct comparison of the two methods in the aspects of the CDR3 AA sequence and V-J gene pairing, especially the latter, clearly illustrated the existence of a method-specific pattern. To determine whether the method variation was more significant than the biological variation among individuals, we compared the method variation with the biological variation between two donors using the same methodology. Using 5’RACE or MPCR, the overlapping rate of the CDR3 AA sequences between two individuals was very low, i.e., less than 10% (S1C and S1F Fig), which was significantly less than the rate of the same sample with different methods (Fig 6A, 6B and 6C). Moreover, the correlation coefficient (less than 0.017) of the CDR3 sequences between two different donors (S1A and S1D Fig) was also far less than that of the same individual (Fig 6D, 6E and 6F). However, for the VJ pairing, the correlation coefficient between two different individuals, which ranged from 0.3431 to 0.5450 (S1B and S1E Fig), was slightly increased (Fig 7). This finding clearly indicated that although it had an influence, method specificity was not as significant as individual specificity in the TCR sequence level.

### Saturation Analysis and Error Comparison

How the number of clonotypes tended to saturate with the increase in the sequencing amount in a TCR repertoire remained unclear, and the answer would be of interest to researchers. Saturation curves of the samples using MPCR or 5’ RACE are shown (S2 Fig). Sequences were randomly subsampled at an interval of 0.5 million sequences or 1 million sequences. All samples reached saturation with the increase of the effective data, which indicated that the sequencing data size of each sample was sufficient and the analyses were credible. The saturating values were different for the 5’RACE and MPCR samples. More specifically, the number of unique CDR3 that reached the saturation was higher in MPCR compared with 5’RACE when starting with the same amount of RNA. More RNA input was required by 5’RACE to reach the same level of saturation with MPCR, which may be because of the random shearing step in which some clonotypes were lost in 5’RACE.

**Fig 8:**
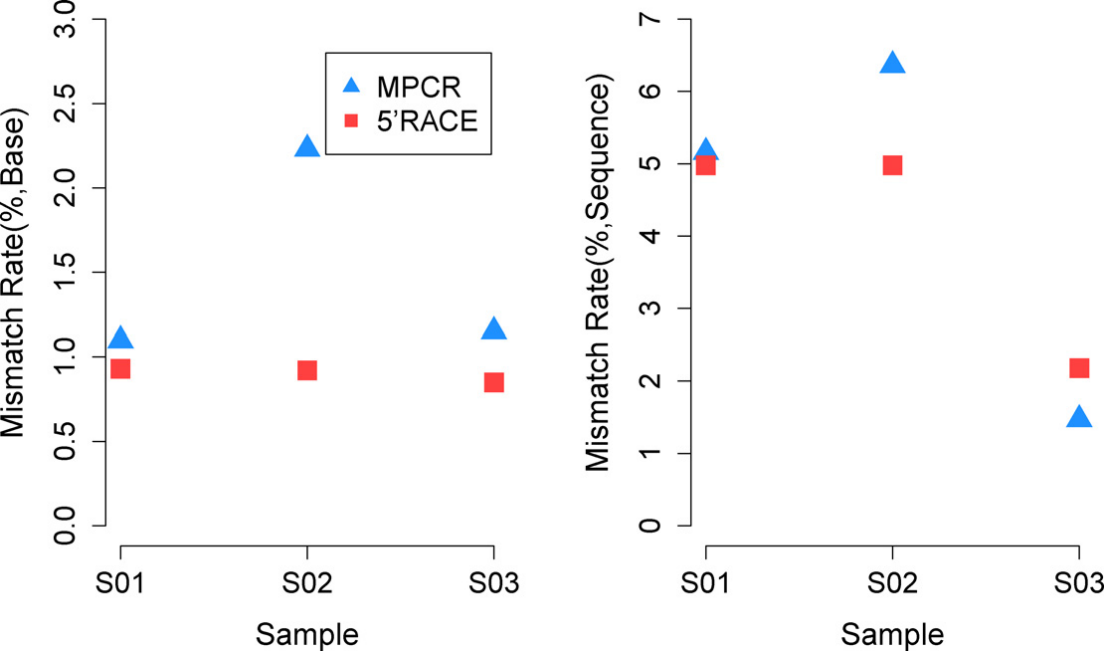
Comparison of PCR/Sequencing errors induced by MPCR and 5’RACE. The mismatch derived from the alignment results of the arrangement sequence to the V/J germline genes and alleles (IMGT). The left indicates the percentage of mismatched nucleotides in total nucleotides (divided by the total bases); the right indicates the rate of the sequences that contain mismatches in total sequences (divided by total sequences).

TCRs do not undergo somatic hyper-mutation in the recombination process; thus, the aligning mismatches with their germline genes were induced by the PCR and sequencing error, together with a certain degree of germline gene polymorphism. We subsequently compared the mismatches of both methods to estimate their errors. As expected, MPCR composed more errors compared with 5’RACE when calculated both by bases and sequences (Fig 8), which was probably contributed to its multiplex amplification.

## Discussion

MPCR and 5’RACE are the most common experimental methods used to investigate the immune repertoire. 5’RACE is less biased [2], whereas the effect of MPCR appears to be influenced by the usage of multiple primers. Therefore, we first evaluated the bias of MPCR using two different control samples. Few templates exhibited bias; moreover, the correlation coefficient between the observed and expected frequencies for the plasmid pools reached 0.9130. The frequencies of five spiked-in clones in three samples were close to the expected frequencies, which was similar with the previous reported[17]. These evaluations demonstrated that although some MPCR primers exhibited certain bias, it was within the acceptable range for it to be used in the subsequent analysis.

Next, the experimental reproducibility of MPCR and 5’RACE was evaluated. It must be emphasized that the two RNA aliquots used as replicates only represent parts of the entire repertoire, which may yield deviation for some TCR clones, especially the low frequency clones. The results demonstrated a strong similarity between the MPCR and 5’RACE samples (correlation coefficients of 0.9907 for MPCR and 0.9878 for 5’RACE) based on their CDR3 amino acid sequence abundance analysis, in which more than 68% of the sequences were identical in the top 1000 CDR3 sequences of the two sorted replicates. The reproducibility of 5’RACE was slightly lower than MPCR, which may be a result of the random shearing step in which only the biotinylated sequences were selected for 5’RACE. This finding also explains a potential reason for the greater difference in the number of unique CDR3 nucleotides and amino acids for 5’RACE. Overall, both methods were highly reproducible with excellent stability.

We identified considerable diversity in the TCRs, as well as biological differences between the divided blood samples or RNA from the same individual. Most V and J genes (~60%) exhibited comparable frequencies between the two methods for the three individuals investigated (Fig 5), with the exception of several genes that exhibited a significant difference. Sixteen V genes and 6 J genes had a significant difference with p-values <0.05, which creates a strong bias for several genes in the MPCR samples. Notably, Freeman et al. described a healthy individual V and J usage with the 5’RACE method, in which the top 3 V genes were TRBV20-1, TRBV5-1, and TRBV29-1, respectively, whereas the top 5 J genes were TRBJ2-1, TRBJ1-1, TRBJ2-7, TRBJ2-3 and TRBJ2-5, respectively. Most of these genes also appeared in high frequency in our samples, which implies a similarity in the V/J gene usage distribution among healthy individuals and the reliability of our method [7].

We demonstrated that there were 17.07–21.07% overlapping unique CDR3 sequences between the MPCR and 5’RACE samples from the same individual (Fig 6). This finding was lower than expected and may be attributed to the biotinylated sequence selection of 5’RACE; the bias of MPCR; the amplification and sequencing errors and the diversity of RNA replicates. Considering the sequence abundance, the overlapping rate increased to an average of 55.42% (S2 Table), which indicated that the corresponding high-frequency sequences had been accurately profiled. The consistency and correlation of the VJ pairing were also calculated (Fig 7). Moreover, the sequences of the 5’RACE samples included nearly all of the V-J pairings, whereas the MPCR samples lost 63 V-J parings on average (Fig 7), which indicates that 5’RACE had a higher efficiency to capture the different clones. As a result of the different RNA amounts used in the two methods for S01/S02, the correlation, consistency and V-J pairing of S01/S02 were worse compared with S03. Furthermore, the consistency and correlation of the CDR3 AA sequence for different samples utilizing the same experimental methods were analyzed, and the methodological differences were compared; however, the latter was demonstrated to be trivial as expected.

In summary, MPCR is a convenient method to enrich the CDR3 region. Notably, the primers designed and adjusted for the MPCR approach influence its performance and the subsequent results of the comparison. The primers adopted in this study were individually optimized for efficient amplification to ensure a satisfactory performance in our pooling plasmids of known clones. However, when tested in biological samples, the performance of several specific genes may remain problematic, such as the lower usage of several J genes and the loss of several V-J parings in MPCR compared with 5’RACE. Our experiments have demonstrated the potential to achieve comparable results between the two methods; however, there are ways to optimize MPCR, such as adjusting the primer efficiency and fine-tuning the ratio of the primers, which is typically difficult. Furthermore, reducing the cycle number of MPCR was an effective way to avoid bias; however, an excessive reduction caused failure to enrich the CDR3 region. To overcome this problem, two-step PCR, in which a universal sequence is added to the 5’ end of the gene specific MPCR primers to enable a reduction in the cycles of the gene specific amplification and subsequent enrichment by the universal primers, was used[26]. In addition, in some research, random barcodes are added to the gene specific MPCR primers to correct the mismatches induced by PCR or sequencing and the amplification bias[26, 27].

5’RACE appeared to be a less biased method to enrich the CDR3 region. In the current setting, the 5’RACE products needed to be subjected to fragmentation and affinity purification, and these procedures result in the loss of low copy transcripts to a certain degree, which causes an inaccurate profiling of the immune repertoire. Therefore, for the 5’RACE immune repertoire libraries, direct sequencing without fragmentation would prove ideal, which could be achieved in other sequencing platforms with longer reads, such as Miseq or 454 sequencer.

Immune repertoire analysis has been widely applied to various fields, such as the monitoring of minimal residue disease for lymphocytic leukemia, vaccine performance evaluation and isolation of monoclonal antibodies. The selection of the proper method and the evaluation of its properties are crucial to scientific discoveries and a better understanding/interpretation of the captured repertoire diversity. For example, extraction of the genuine frequencies of cancer clones in the relapse of leukemia is the key for the application.

Our study provides a direct head-to-head comparison of the two major experimental methodologies commonly used to investigate the immune repertoire. We believe that the systematic evaluation of the platform-specific characteristics and bias would provide useful suggestions for researchers to select the suitable method and diminish the platform-induced bias/errors. One limitation was the use of Hiseq 2000, which was constraining because of the short read lengths that resulted from this platform. A similar constraint is associated with standard 5’RACE, which cannot be investigated without the fragmentation of cDNA and has been selected by other studies performed in combination with 454 sequencer. Overall, considering that we used the most widely used sequencing platform, the current findings will be of value to immunology research and will benefit the development of related applications in the field.

## Supporting Information

S1 Fig Comparison of different individuals with the same method. (**a**). The correlation of the CDR3 AA sequences between samples S01 and S02 using 5’RACE. Each dot represents a unique CDR3 AA sequence. (**b**). The correlation of VJ pairing between samples S01 and S02 using 5’RACE. Each dot represents a unique type of VJ pairing. (**c**). The consistency of the CDR3 AA sequences between samples S01 and S02 using 5’RACE. Black circles represent the overlapping rate of the two sorted data sets; grey circles represent the number of overlapping sequences of the two sorted data sets. In (**a**) and (**b**), the number in the top left corner indicates the Pearson correlation coefficient of the two data sets. (**d**). The correlation of the CDR3 AA sequences between samples S01 and S02 using MPCR. (**e**). The correlation of the VJ pairing between samples S01 and S02 using MPCR. (**f**). The consistency of the CDR3 AA sequences between samples S01 and S02 using MPCR. Black, overlapping rate; Grey, number of overlapped sequences.
(DOCX)

S2 Fig Saturation analysis for the three individuals. The saturation curve was plotted for each individual using the observed and predicted (Chao1) numbers of unique CDR3 AA for both MPCR and 5’RACE. **(a)**. Sample S01. **(b)**. Sample S02. **(c)**. Sample S03. **(d)**. Saturation value distribution for all samples.
(DOCX)

S1 Table Basic statistics of two repetitive MPCR and 5'RACE libraries. (DOCX)

S2 Table Comparison of two methods for 3 individuals. (DOCX)

S3 Table Plasmid mix pattern. (DOCX)

S4 Table Experimental design for five CD4+ T cell clones. (DOCX)
