# Supplementary material for: Systematic Comparative Evaluation of Methods for Investigating the TCRβ Repertoire
Source: PLoS One. 2016 Mar 28;11(3):e0152464. doi: 10.1371/journal.pone.0152464 (PMC4809601; doi:10.1371/journal.pone.0152464)
Supplement: S1 Fig — (a). The correlation of the CDR3 AA sequences between samples S01 and S02 using 5’RACE. Each dot represents a unique CDR3 AA sequence. (b). The correlation of VJ pairing between samples S01 and S02 using 5’RACE. Each dot represents a unique type of VJ pairing. (c). The consistency of the CDR3 AA sequences between samples S01 and S02 using 5’RACE. Black circles represent the overlapping rate of the two sorted data sets; grey circles represent the number of overlapping sequences of the two sorted data sets. In (a) and (b), the number in the top left corner indicates the Pearson correlation coefficient of the two data sets. (d). The correlation of the CDR3 AA sequences between samples S01 and S02 using MPCR. (e). The correlation of the VJ pairing between samples S01 and S02 using MPCR. (f). The consistency of the CDR3 AA sequences between samples S01 and S02 using MPCR. Black, overlapping rate; Grey, number of overlapped sequences. (DOCX) [file pone.0152464.s001.docx]

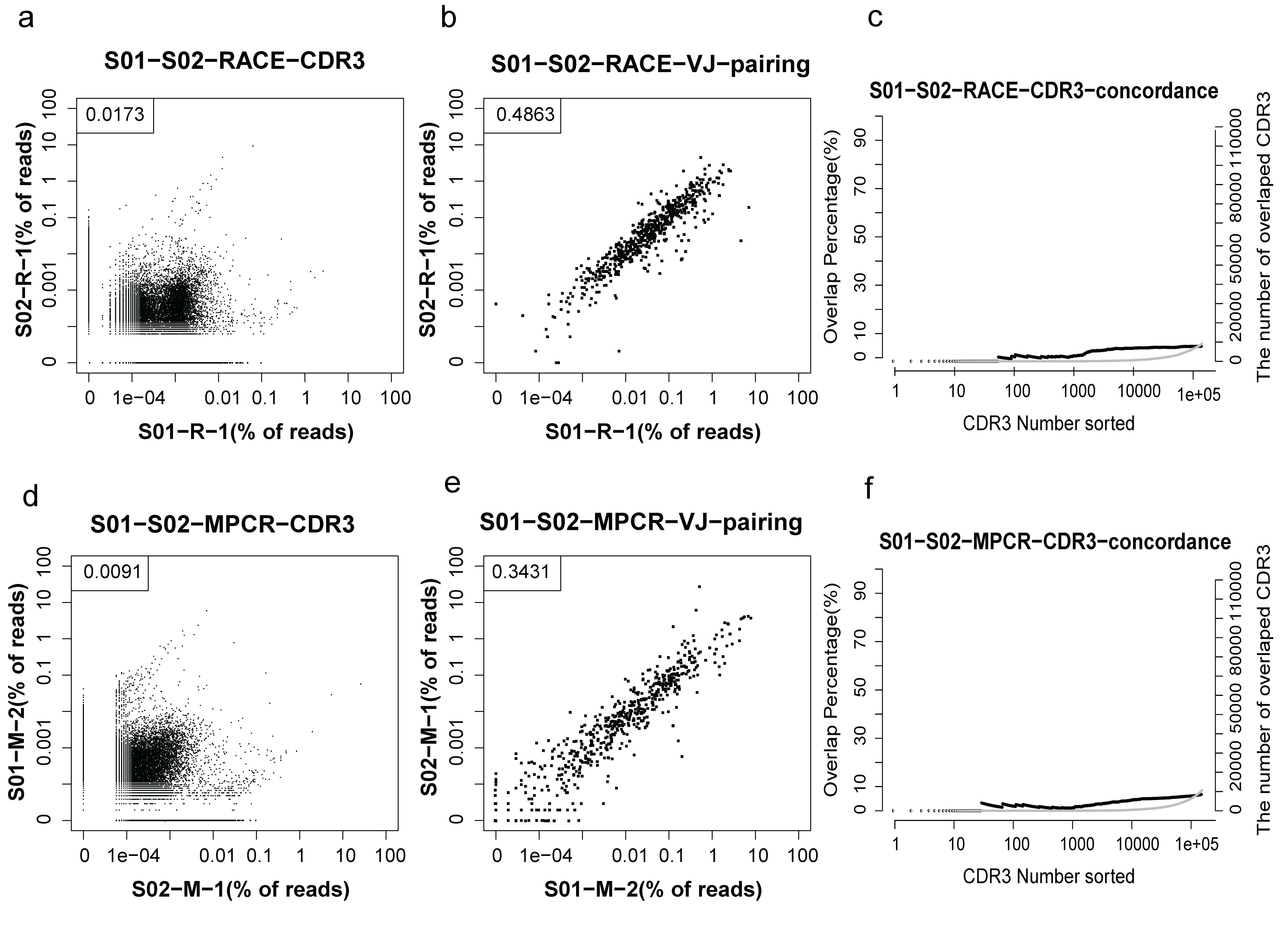


**S1 Fig. Comparison of different individuals with the same method.** (**a**). The correlation of the CDR3 AA sequences between samples S01 and S02 using 5’RACE. Each dot represents a unique CDR3 AA sequence. (**b**). The correlation of VJ pairing between samples S01 and S02 using 5’RACE. Each dot represents a unique type of VJ pairing. (**c**). The consistency of the CDR3 AA sequences between samples S01 and S02 using 5’RACE. Black circles represent the overlapping rate of the two sorted data sets; grey circles represent the number of overlapping sequences of the two sorted data sets. In (**a**) and (**b**), the number in the top left corner indicates the Pearson correlation coefficient of the two data sets. (**d**). The correlation of the CDR3 AA sequences between samples S01 and S02 using MPCR. (**e**). The correlation of the VJ pairing between samples S01 and S02 using MPCR. (**f**). The consistency of the CDR3 AA sequences between samples S01 and S02 using MPCR. Black, overlapping rate; Grey, number of overlapped sequences..
