# Supplementary material for: Systematic Comparative Evaluation of Methods for Investigating the TCRβ Repertoire
Source: PLoS One. 2016 Mar 28;11(3):e0152464. doi: 10.1371/journal.pone.0152464 (PMC4809601; doi:10.1371/journal.pone.0152464)
Supplement: S2 Fig — The saturation curve was plotted for each individual using the observed and predicted (Chao1) numbers of unique CDR3 AA for both MPCR and 5’RACE. (a). Sample S01. (b). Sample S02. (c). Sample S03. (d). Saturation value distribution for all samples. (DOCX) [file pone.0152464.s002.docx]

**
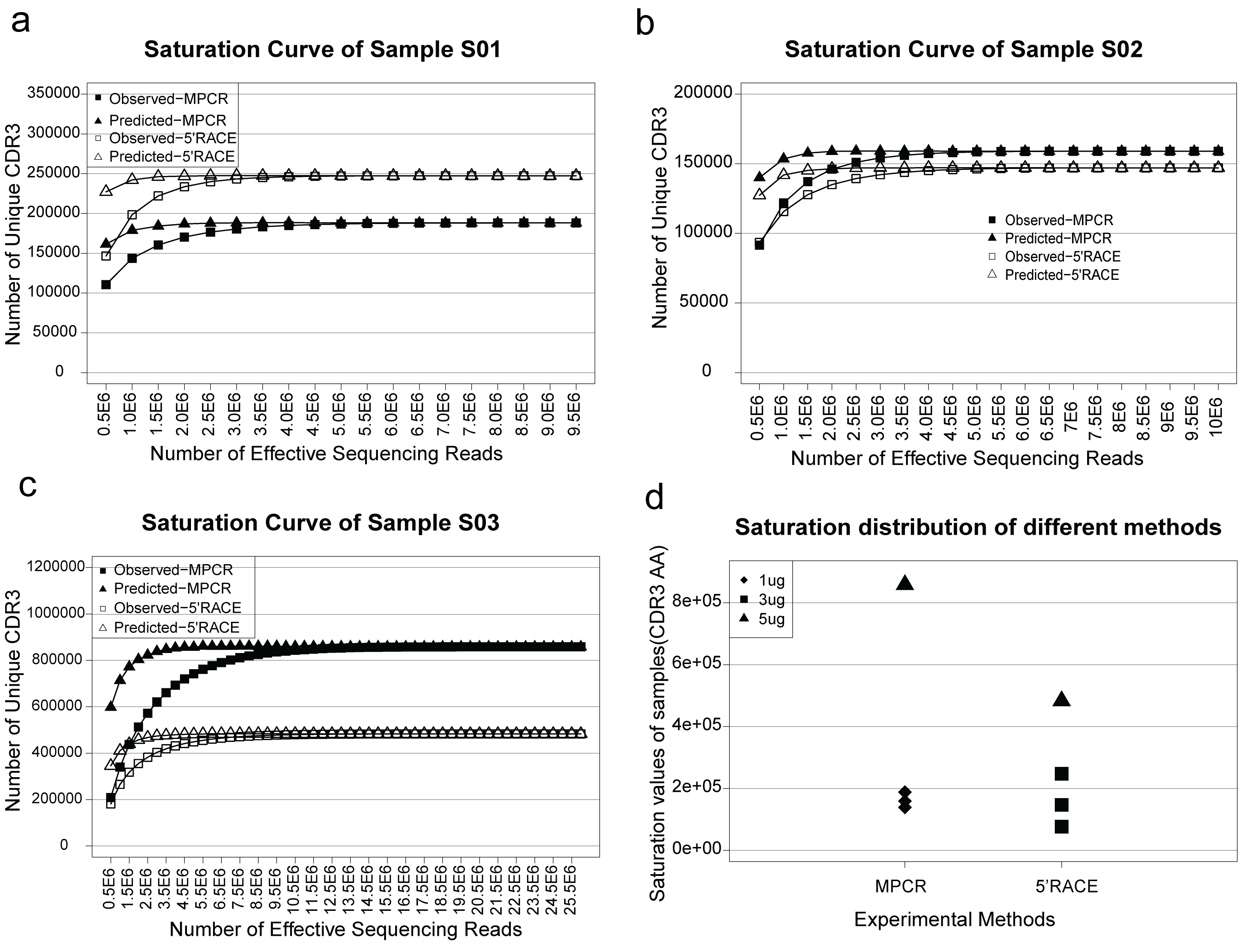
**

**S2 Fig. Saturation analysis for the three individuals.** The saturation curve was plotted for each individual using the observed and predicted (Chao1) numbers of unique CDR3 AA for both MPCR and 5’RACE. **(a)**. Sample S01. **(b)**. Sample S02. **(c)**. Sample S03. **(d)**. Saturation value distribution for all samples.
