# Supplementary material for: Systematic Comparative Evaluation of Methods for Investigating the TCRβ Repertoire
Source: PLoS One. 2016 Mar 28;11(3):e0152464. doi: 10.1371/journal.pone.0152464 (PMC4809601; doi:10.1371/journal.pone.0152464)
Supplement: S1 Table — (DOCX) [file pone.0152464.s003.docx]

| **S1 Table. Basic statistics of two repetitive MPCR and 5'RACE libraries** | | | | |
| --- | --- | --- | --- | --- |
|  | **S01-M-1** | **S01-M-2** | **S02-R-1** | **S02-R-2** |
| **Effective TCRs** | 6,880,594 | 6,880,594 | 11,023,329 | 11,023,329 |
| **Simpson's diversity index(CDR3 AA*)** | 0.9961 | 0.9949 | 0.9985 | 0.9985 |
| **Unique CDR3 Nucleotide** | 148,262 | 203,828 | 156,655 | 81,008 |
| **Unique CDR3 AA** | 138,831 | 187,975 | 147,035 | 76,636 |
| **Shared unique CDR3 AA** | 69,634 | 69,634 | 25,773 | 25,773 |
|  | 50.16% | 37.04% | 17.53% | 33.63% |
| **Reads with shared CDR3** | 5,410,329 | 5,155,925 | 5512767 | 6578723 |
|  | 78.63% | 74.93% | 50.01% | 59.68% |
| **R2(Pearson’s correlation coefficient)** | 0.9907 | | 0.9878 | |
| *: AA, Amino Acid. A unique CDR3 sequence means a fragment of amino acids or nucleic acids different with any others | | | | |
