# Supplementary material for: Systematic Comparative Evaluation of Methods for Investigating the TCRβ Repertoire
Source: PLoS One. 2016 Mar 28;11(3):e0152464. doi: 10.1371/journal.pone.0152464 (PMC4809601; doi:10.1371/journal.pone.0152464)
Supplement: S2 Table — (DOCX) [file pone.0152464.s004.docx]

| **S2 Table. Comparison of two methods for 3 individuals** | | | | |
| --- | --- | --- | --- | --- |
| **Sample** | **Method** | **Effective sequences** | **Overlapped sequences*** | **Overlap Rate** |
| **S01-M-2** | **MPCR** | 9,585,671 | 5,693,852 | 59.40% |
| **S01-R-1** | **5'RACE** |  | 5,185,496 | 54.10% |
| **S02-M-1** | **MPCR** | 10,398,513 | 6,895,776 | 66.32% |
| **S02-R-1** | **5'RACE** |  | 4,474,324 | 43.03% |
| **S03-M-1** | **MPCR** | 26,067,794 | 14,592,947 | 55.98% |
| **S03-R-1** | **5'RACE** |  | 13,996,249 | 53.69% |
| *: Overlapped sequences were the sequences with the same CDR3 AA between MPCR and 5’RACE | | | | |
