# Supplementary material for: Systematic Comparative Evaluation of Methods for Investigating the TCRβ Repertoire
Source: PLoS One. 2016 Mar 28;11(3):e0152464. doi: 10.1371/journal.pone.0152464 (PMC4809601; doi:10.1371/journal.pone.0152464)
Supplement: S3 Table — (DOCX) [file pone.0152464.s005.docx]

| **S3 Table. Plasmid mix pattern.** | | | | |  |
| --- | --- | --- | --- | --- | --- |
| **Plasmid No.** | **V gene** | **J gene** | **Plasmid mix 1(pmole)** | **Plasmid mix 2(pmole)** | **Plasmid mix 3(pmole)** |
| C-01 | TRBV10-1 | TRBJ2-7 | 2000 | 10 | 100000 |
| C-02 | TRBV10-2/3 | TRBJ2-7 | 2000 | 1000 | 1000 |
| C-03 | TRBV11-1/2/3 | TRBJ1-3 | 2000 | 10 | 100000 |
| C-04 | TRBV11-1/2/3 | TRBJ1-5 | 2000 | 100 | 10000 |
| C-05 | TRBV12-3/4 | TRBJ2-1 | 2000 | 10000 | 100 |
| C-06 | TRBV12-5 | TRBJ2-1 | 2000 | 100 | 10000 |
| C-07 | TRBV13 | TRBJ1-1 | 2000 | 10000 | 100 |
| C-08 | TRBV14 | TRBJ2-7 | 2000 | 100000 | 10 |
| C-09 | TRBV15 | TRBJ1-6 | 2000 | 1000 | 1000 |
| C-10 | TRBV15 | TRBJ2-4 | 2000 | 100000 | 10 |
| C-11 | TRBV16 | TRBJ1-1 | 2000 | 100000 | 10 |
| C-12 | TRBV19 | TRBJ1-6 | 2000 | 100 | 10000 |
| C-13 | TRBV20-1 | TRBJ1-4 | 2000 | 100000 | 10 |
| C-14 | TRBV20-1 | TRBJ1-5 | 2000 | 10 | 100000 |
| C-15 | TRBV20-1 | TRBJ2-2 | 2000 | 1000 | 1000 |
| C-16 | TRBV24-1 | TRBJ1-2 | 2000 | 100 | 10000 |
| C-17 | TRBV25 | TRBJ2-5 | 2000 | 10000 | 100 |
| C-18 | TRBV27/28 | TRBJ2-4 | 2000 | 100000 | 10 |
| C-19 | TRBV29-1 | TRBJ2-3 | 2000 | 100000 | 10 |
| C-20 | TRBV2 | TRBJ2-6 | 2000 | 10 | 100000 |
| C-21 | TRBV30 | TRBJ1-1 | 2000 | 1000 | 1000 |
| C-22 | TRBV3-1 | TRBJ1-2 | 2000 | 10000 | 100 |
| C-23 | TRBV4-1/2/3 | TRBJ2-7 | 2000 | 10000 | 100 |
| C-24 | TRBV5-1 | TRBJ2-1 | 2000 | 100000 | 10 |
| C-25 | TRBV5-4/5/6/8 | TRBJ2-3 | 2000 | 10000 | 100 |
| C-26 | TRBV6-1/2/3/5/8 | TRBJ2-1 | 2000 | 10000 | 100 |
| C-27 | TRBV6-4 | TRBJ2-5 | 2000 | 1000 | 1000 |
| C-28 | TRBV6-6 | TRBJ1-6 | 2000 | 10 | 100000 |
| C-29 | TRBV6-9 | TRBJ1-3 | 2000 | 1000 | 1000 |
| C-30 | TRBV7-2/4/6/7/8 | TRBJ2-6 | 2000 | 10 | 100000 |
| C-31 | TRBV7-3 | TRBJ2-7 | 2000 | 100 | 10000 |
| C-32 | TRBV7-9 | TRBJ1-4 | 2000 | 1000 | 1000 |
| C-33 | TRBV9 | TRBJ1-2 | 2000 | 100 | 10000 |
