# Supplementary material for: Systematic Comparative Evaluation of Methods for Investigating the TCRβ Repertoire
Source: PLoS One. 2016 Mar 28;11(3):e0152464. doi: 10.1371/journal.pone.0152464 (PMC4809601; doi:10.1371/journal.pone.0152464)
Supplement: S4 Table — (DOCX) [file pone.0152464.s006.docx]

**S4 Table. Experimental design for five CD4+ T cell clones**

| **Clone** | **TCRB V** | **TCRB J** | **CDR3** | **Mix 1** | **Mix 2** | **Mix 3** |
| --- | --- | --- | --- | --- | --- | --- |
| **G** | VB8 | TRBJ1-1 | CASSLGGQGVG | 100,000 | 1000 | 10 |
| **A** | VB5.1 | TRBJ2-5 | CASSPGIAELKETQY | 10,000 | 1000 | 100 |
| **B** | VB6.7 | TRBJ2-7 | CASHTGFVSYEQY | 1000 | 1000 | 1000 |
| **C** | VB4 | TRBJ1-4 | CSVGTGDNEKLF | 100 | 1000 | 10,000 |
| **D** | VB4 | TRBJ1-4 | CSVGQGDNEKLF | 10 | 1000 | 100,000 |
